# Effect and limitation of neoadjuvant chemotherapy for pancreatic ductal adenocarcinoma: consideration from a new perspective

**DOI:** 10.1186/s12957-021-02192-8

**Published:** 2021-03-22

**Authors:** Yoshihiro Kurata, Takayuki Shiraki, Masanori Ichinose, Keiichi Kubota, Yasuo Imai

**Affiliations:** 1 Department of Surgery, Chiba University Hospital, Chiba, Japan; 2 Department of Surgery, Shioya Hospital, International University of Health and Welfare, Tochigi, Japan; 3 Department of Gastroenterological Surgery, Dokkyo Medical University, Tochigi, Japan; 4 Department of Diagnostic Pathology, Ota Memorial Hospital, SUBARU Health Insurance Society, 455-1 Oshima, Gunma, 373-8585 Japan

**Keywords:** Pancreatic ductal adenocarcinoma, Neoadjuvant chemotherapy, Gemcitabine, S-1, GS, Upfront surgery, Prognosis, Downstaging, Micro-metastasis

## Abstract

**Background:**

Effect of neoadjuvant chemotherapy (NAC) for pancreatic ductal adenocarcinoma (PDAC) has remained under investigation. We investigated its effect from a unique perspective and discussed its application.

**Patients and methods:**

We retrospecively analyzed consecutive 131 PDAC patients who underwent pancreatoduodenectomy and distal pancreatectomy. Clinicopathologic data at surgery and postoperative prognosis were compared between patients who underwent upfront surgery (UFS) (*n* = 64) and those who received NAC (*n* = 67), of which 62 (92.5%) received gemcitabine plus S-1 (GS). The GS regimen resulted in about 15% of partial response and 85% of stable disease in a previous study which analyzed a subset of this study subjects.

**Results:**

Tumor size was marginally smaller, degree of nodal metastasis and rate of distant metastasis were significantly lower, and pathologic stage was significantly lower in the NAC group than in the UFS group. In contrast, significant differences were not observed in histopathologic features such as vessel and perineural invasions and differentiation grade. Notably, disease-free and overall survivals were similar between the two groups adjusted for the pathologic stage, suggesting that effects of NAC, including macroscopically undetectable ones such as control of micro-metastasis and devitalizing tumor cells, may not be remarkable in the majority of PDAC, at least with respect to the GS regimen.

**Conclusions:**

NAC may be useful in downstaging and improving prognosis in a small subset of tumors. However, postoperative prognosis may be determined at the pathologic stage of resected specimen with or without NAC. Therefore, NAC may be applicable to borderline resectable and locally advanced PDAC for enabling surgical resection, but UFS would be desirable for primary resectable PDAC.

## Introduction

Pancreatic ductal adenocarcinoma (PDAC) is one of the most lethal cancers with an overall 5-year survival rate of only 9% for all stages combined [1]. Surgery offers the best chance for long-term survival, but the majority of patients have either locally extended tumor growth or distant tumor spread and resection is possible in only 15–20% of all patients [2]. PDAC is classified as resectable, borderline resectable, and locally advanced at the time of initial diagnosis. Briefly, resectable PDAC does not contact with the muscular artery (celiac axis, superior mesenteric artery, and hepatic artery) at all and vein (superior mesenteric vein (SMV) and portal vein (PV)) of > 180° or with vein contour irregularity. Locally advanced PDAC contacts with the aforementioned muscular artery of > 180° or aorta or with SMV or PV which cannot be reconstructed. The other PDAC is classified as borderline resectable [3]. Resectable PDAC is an indication of upfront surgery (UFS), and neoadjuvant chemotherapy (NAC) is not encouraged [4]. In contrast, borderline resectable and locally advanced PDACs are not candidates for UFS and NAC is usually performed to downsize tumors and enable R0 resection (microscopically invasive cancer-free at all margins) [4]. Resection rate of borderline resectable PDAC was reported to be 65.3% after NAC [5]. Another study reported that 46 to 61% of locally advanced and unresectable PDAC could undergo resection after NAC with or without other treatments [6]. Thus, NAC may improve prognosis in approximately 60% of patients with borderline resectable and locally advanced PDAC by enabling surgical resection. NAC is also intended to treat micro-metastasis and devitalize tumors even if imaging studies do not suggest downsizing and to select candidates for radical resection. The last purpose is based on the theory that PDAC which shows progression during a short timeframe of NAC would be biologically aggressive. Thus far, effects of neoadjuvant treatment have been analyzed by retrospective studies, which included many limitations [5, 7, 8]. The most ideal study design would be a prospective one to compare survival times of randomly assigned patients between the date of initial diagnosis or randomization and the date of event or last known contact in intention-to-treat analysis. However, recruiting patients for such clinical studies may be difficult or get suspended because of the aggressive nature of the disease [4]. In this study, we investigated the effect of NAC from a different approach. In Japan, a combination of gemcitabine plus S-1 (tegafur/gimeracil/oteracil) (GS) was employed as standard for patients with advanced PDAC until the clinical introduction of 5-fluorouracil/leucovorin plus irinotecan plus oxaliplatin (FOLFIRINOX) or gemcitabine plus nab-paclitaxel (GnP) therapy [9–11]. Neoadjuvant GS is also attracting attention in resectable and borderline resectable PDAC (PREP-01 and PREP-02 studies) [12, 13]. By comparing postoperative prognosis adjusted for the pathologic stage, we attempted to draw useful information on the clinical effect and indication of NAC, especially GS regimen.

## Materials and methods

### Patients

We retrospectively analyzed consecutive patients who underwent surgery for PDAC at Dokkyo Medical University Hospital between 2011 and 2018 and at Shioya Hospital, International University of Health and Welfare, between 2006 and 2019. All patients, either following NAC or not, underwent surgery with curative intent. Fourteen cases of total pancreatectomy and seven cases complicated by malignancies in other organs were excluded. As a result, a total of 131 patients were included in this study. Patients’ clinicopathologic data were obtained via the electric medical chart system in each institution. After surgery, patient follow-up was performed every month at the outpatient clinic for 5 years after surgery or until they were referred to other institutions for social reasons or deteriorated performance status. A blood test was performed every 2 months, and radiographic imaging studies were performed every 3 months for the first 6 months, every 6 months for 18 more months, and yearly for 3 more years. Postoperative recurrence and metastasis were detected mostly by biochemical markers and radiographic modalities. Diagnoses of peritoneal and pleural metastases were performed by cytological investigation. Local recurrence was defined as the appearance of new mass lesions by contrast-enhanced computed tomography (CT), magnetic resonance imaging, or positron emission tomography-CT within the resection field and pancreatojejunal anastomosis site where surgeons thought that tumors could be removed without macroscopic remnants. Therefore, diagnosis of the local recurrence was made irrespective of pathologic resection margin status, whether it was R0 or R1 (microscopically involved by invasive cancer at any margin). This definition of local recurrence is in accordance with that by Gnerlich et al. [14]. Metastases to other organs and recurrence in non-regional lymph nodes were categorized as distant metastasis. Re-elevated biochemical markers after surgery without a mass lesion recognizable by imaging modalities were judged as distant metastasis to an unknown site. Disease-free survival times were those without macroscopically residual invasive cancer, local recurrence, or distant metastasis. Histopathologic diagnosis was performed using the World Health Organization classification of Tumours of the Digestive System, 4th edition [15], and stage grouping was performed according to the TNM Classification of Malignant Tumors, 8th edition [16]. The study protocol was approved by the institutional ethics review boards of both institutions (approvals R-12-20J and 13-B-316).

### Statistical analysis

Clinicopathologic data obtained at the time of surgery were analyzed. Specific parameters between two patient cohorts and associations between two variables were compared using Fisher’s exact test except for age, which was compared using the Mann–Whitney *U* test. Survival curves were generated using the Kaplan-Meier method, and curves were compared by the log-rank test. Statistical analysis was performed using IBM SPSS Statistics 25 (IBM, Armonk, NY, USA).

## Results

### Clinicopathologic findings

The patients consisted of 72 (55.0%) males and 59 (45.0%) females aged 43 to 90 years old, with a median age of 69, and 79 (60.3%) had PDAC in the pancreatic head and 52 (39.7%) had PDAC in the body and tail at surgery. NAC, consisting of gemcitabine, S-1, and/or nab-paclitaxel was performed in 67 (51.1%) patients. Out of the 67 patients, 40 patients had resectable PDAC and 20 patients had borderline resectable or locally advanced PDAC. Information about resectability was not available in 7 patients. The breakdown of NAC regimen is as follows: gemcitabine in two patients, GS in 62 patients, GnP in two patients, and GnP following GS in one patient. Thus, 92.5% of NAC is the two cycles of GS regimen, the detail of which is described elsewhere [17]. Cycles of other NAC regimen was flexibly determined in accordance with the patient’s general condition. Pancreatoduodenectomy and distal pancreatectomy were performed in 79 (60.3%) and 52 (39.7%) patients, respectively. Pathologic examination of resected specimens revealed that 49 (37.4%), 55 (42.0%), 22 (16.8%), and 5 (3.8%) patients were stage I, II, III, and IV, respectively. Postoperative chemotherapy (adjuvant chemotherapy and chemotherapy for recurrence/metastasis), consisting of gemcitabine, S-1, cisplatin (CDDP) plus gemcitabine or S-1, paclitaxel monotherapy, erlotinib plus gemcitabine, GS, GnP, or FOLFIRINOX was performed in 113 (86.3%) patients. All patients who received postoperative chemotherapy started with gemcitabine or S-1 monotherapy and were treated up to the 5th line with different drug types. Seven (5.3%) patients received irradiation for the treatment of local recurrence or distant metastasis postoperatively. The patients’ clinicopathological findings are summarized in Table 1.

**Table 1:** Clinicopathologic features of the 131 patients at the time of surgery

Parameters	Total (*n* = 131)	UFS (*n* = 64)	NAC (*n* = 67)	NAC (GS) (*n* = 62)	*P* value (UFS *v.s.* NAC)	*P* value (UFS *v.s.* NAC (GS))
Median age (range)	69 (43-90)	69 (46-90)	69 (43-84)	68 (43-84)	0.163	0.105
Sex						
Male	72	38	34	32	0.381	0.473
Female	59	26	33	30		
Date of surgery						
Before 2014	42	39	3	1	< 0.001	< 0.001
2014 and later	89	25	64	61		
Tumor location						
Head	79	43	36	34	0.153	0.201
Body and tail	52	21	31	28		
Tumor size						
pT1	25	8	17	16	0.076	0.071
pT2/pT3	106	56	50	46		
Tumor size						
pT1/pT2	113	55	58	54	1.000	1.000
pT3	18	9	9	8		
Differentiation grade						
G1/G2	112	52	60	55	0.218	0.321
G3/G4	19	12	7	7		
Differentiation grade						
G1	56	27	29	27	1.000	1.000
G2/G3/G4	75	37	38	35		
Microvascular invasion						
Positive	115	59	56	51	0.397	0.261
Negative	14	5	9	9		
Unknown	2	0	2	2		
Lymphatic permeation						
Positive	96	49	47	43	0.436	0.424
Negative	35	15	20	19		
Perineural invasion						
Positive	111	56	55	50	0.470	0.336
Negative	20	8	12	12		
Nodal metastasis						
pN0	50	20	30	28	0.015	0.005
pN1	57	26	31	30		
pN2	24	18	6	4		
Distant metastasis						
Yes	5	5	0	0	0.026	0.058
No	126	59	67	62		
TNM stage						
I/II	104	43	61	58	0.001	< 0.001
III/IV	27	21	6	4		
R0 resection						
Yes	80	35	45	44	0.156	0.067
No	51	29	22	18		
R2 resection						
Yes	1	0	1	1	1.000	0.492
No	130	64	66	61		
Surgery-related death						
Yes	2	2	0	0	0.237	0.496
No	129	62	67	62		
Postoperative chemotherapy						
Yes	113	54	59	54	0.271	0.399
No	14	9	5	5		
Unknown	4	1	3	3		
Postoperative radiotherapy						
Yes	7	3	4	4	1.000	0.715
No	124	61	63	58		

*NAC* neoadjuvant chemotherapy, *UFS* upfront surgery, *GS* gemcitabine plus S-1 (tegafur/gimeracil/oteracil)*, G1* well differentiated carcinoma, *G2* moderately differentiated carcinoma, *G3* poorly differentiated carcinoma, *G4* undifferentiated carcinoma, *R0* microscopically invasive cancer-free, *R2* macroscopically residual tumor

The clinicopathologic parameters at surgery were compared between patients who underwent UFS and patients who underwent surgery following NAC. NAC came to be commonly performed since 2014 in our institutions. Tumor size measured by pathological investigation was marginally smaller in the NAC group than in the UFS group as shown by pT1 versus pT2/pT3 (*P* = 0.076). There was no significant difference in the differentiation grade, and lymphovascular and neural invasions between the UFS group and the NAC group. However, degree of nodal metastasis was significantly lower (*P* = 0.015) and rate of distant metastasis found at laparotomy was significantly lower (*P* = 0.026) in the NAC group than in the UFS group. As a result, pathologic stages I/II were significantly more frequent than pathologic stages III/IV in the NAC group compared with the UFS group (*P* = 0.001). There were no significant differences in the frequencies of overall postoperative chemotherapy and postoperative radiotherapy between the NAC group and the UFS group. These results are summarized in Table 1. Then, we compared postoperative chemotherapy regimens between the UFS and NAC groups. There were no significant differences in the use of gemcitabine (monotherapy or in combined use), S-1 (monotherapy or in combined use), erlotinib plus gemcitabine, paclitaxel (monotherapy), and GS between the groups, while CDDP plus gemcitabine or S-1 was significantly more frequent in the UFS group but GnP and FOLFIRINOX were significantly more frequent in the NAC group (Table 2).

**Table 2:** Comparison of postoperative chemotherapy between the UFS and NAC groups

Postoperative chemotherapy	Total (*n* = 131)	UFS (*n* = 64)	NAC (*n* = 67)	NAC (GS) (*n* = 62)	*P* value* (UFS *v.s.* NAC)	*P* value* (UFS *v.s.* NAC (GS))
Gemcitabine (monotherapy or combination therapy)	80	39	41	36	0.855	1.000
S-1 (monotherapy or combination therapy)	93	45	48	45	0.692	0.681
CDDP plus gemcitabine or S-1	6	6	0	0	0.013	0.028
Erlotinib plus gemcitabine	2	2	0	0	0.244	0.496
Paclitaxel (monotherapy)	1	1	0	0	1.000	0.496
GS	7	4	3	3	0.718	1.000
GnP	40	14	26	23	0.035	0.051
FOLFIRINOX	12	2	10	10	0.030	0.014

*UFS* upfront surgery, *NAC* neoadjuvant chemotherapy, *S-1* tegafur/gimeracil/oteracil, *GS* gemcitabine plus S-1, *CDDP* cisplatin, *GnP* gemcitabine plus nab-paclitaxel, *FOLFIRINOX* 5-fluorouracil, leucovorin, irinotecan, oxaliplatin

*Cases without information are excluded from calculation of *P* values

### Prognosis

The overall follow-up periods from surgery to cancer-related death or censoring were 20 to 2772 days, with a median of 505 days. Local recurrence was observed in 23 (17.6%) of 131 patients. Distant metastasis was observed in 81 (61.8%) patients: the liver in 33 (25.2%), the lung in 14 (10.7%), the pleura in 5 (3.8%), the peritoneum in 23 (17.6%), the non-regional lymph node in 15 (11.5%), the bone in two (1.5%), and an unknown site in three (2.3%) patients. Local recurrence and/or distant metastasis were observed in a total of 93 (71.0%) patients. PDAC-related death was observed in 82 (62.6%) patients, of which two (1.5%) were caused by surgical complications. One patient died of bleeding 33 days after surgery, and one patient died of bleeding and ensuing liver failure 20 days after surgery. The survival time of patients who died of PDAC itself or surgical complication was 20 to 1435 days, with a median of 442 days.

Surgery-related death was noted in two patients of the UFS group, and R2 resection (macroscopically residual tumor at any margin) was noted in one patient of the NAC group. There was no significant difference in surgery-related death and R2 resection between the UFS group and the NAC group.

**Fig. 1:**
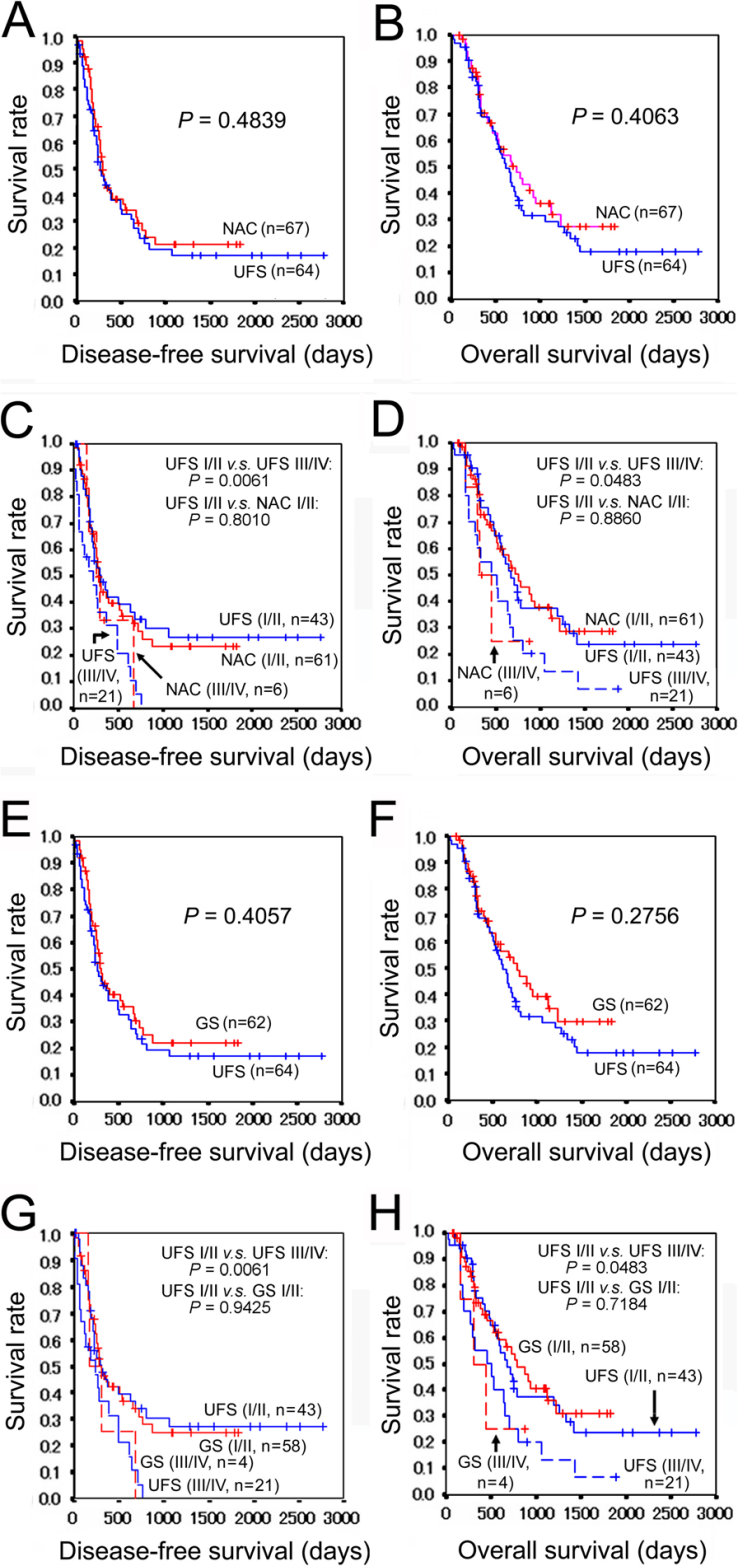
Survival curves of patients with PDAC. **a** Disease-free survivals of the UFS and NAC groups. **b** Overall survivals of the UFS and NAC groups. **c** Comparison of the disease-free survivals between the UFS and NAC groups adjusted for the pathologic stage. **d** Comparison of the overall survivals between the UFS and NAC groups adjusted for the pathologic stage. **e** Disease-free survivals of the UFS and NAC (GS) groups. **f** Overall survivals of the UFS and NAC (GS) groups. **g** Comparison of the disease-free survivals between the UFS and NAC (GS) groups adjusted for the pathologic stage. **h** Comparison of the overall survivals between the UFS and NAC (GS) groups adjusted for the pathologic stage. PDAC, pancreatic ductal adenocarcinoma; UFS, upfront surgery; NAC, neoadjuvant chemotherapy; GS, gemcitabine plus S-1 (tegafur/gimeracil/oteracil)

Postoperative survival curves were drawn and compared between the UFS group and the NAC group. There were no significant differences between the two groups in a disease-free survival (*P* = 0.4839) and an overall survival (*P* = 0.4063), although the overall survival of the NAC group seemed somewhat better than that of the UFS group (Fig. 1a, b).

Next, each group of patients was subdivided according to the pathologic stage. Out of the UFS group, 43 were stages I/II and 21 were stages III/IV. As we expected, patients with stages I/II demonstrated significantly better disease-free and overall survivals than those with stages III/IV (*P* = 0.0061 and 0.0483, respectively) (Fig. 1c, d). Out of the NAC group, 61 were stages I/II and 6 were stages III/IV. Patients with stages I/II tended to show more favorable disease-free and overall survivals than those with stages III/IV, but it was inappropriate to perform statistical analysis because of the small number of patients with stages III/IV. (Fig. 1c, d) Then, prognoses of both groups were compared with the stages adjusted. The UFS group with stages I/II (*n* = 43) and the NAC group with stages I/II (*n* = 61) demonstrated similar disease-free survivals (*P* = 0.8010) and overall survivals (*P* = 0.8860) (Fig. 1c, d).

**Fig. 2:**
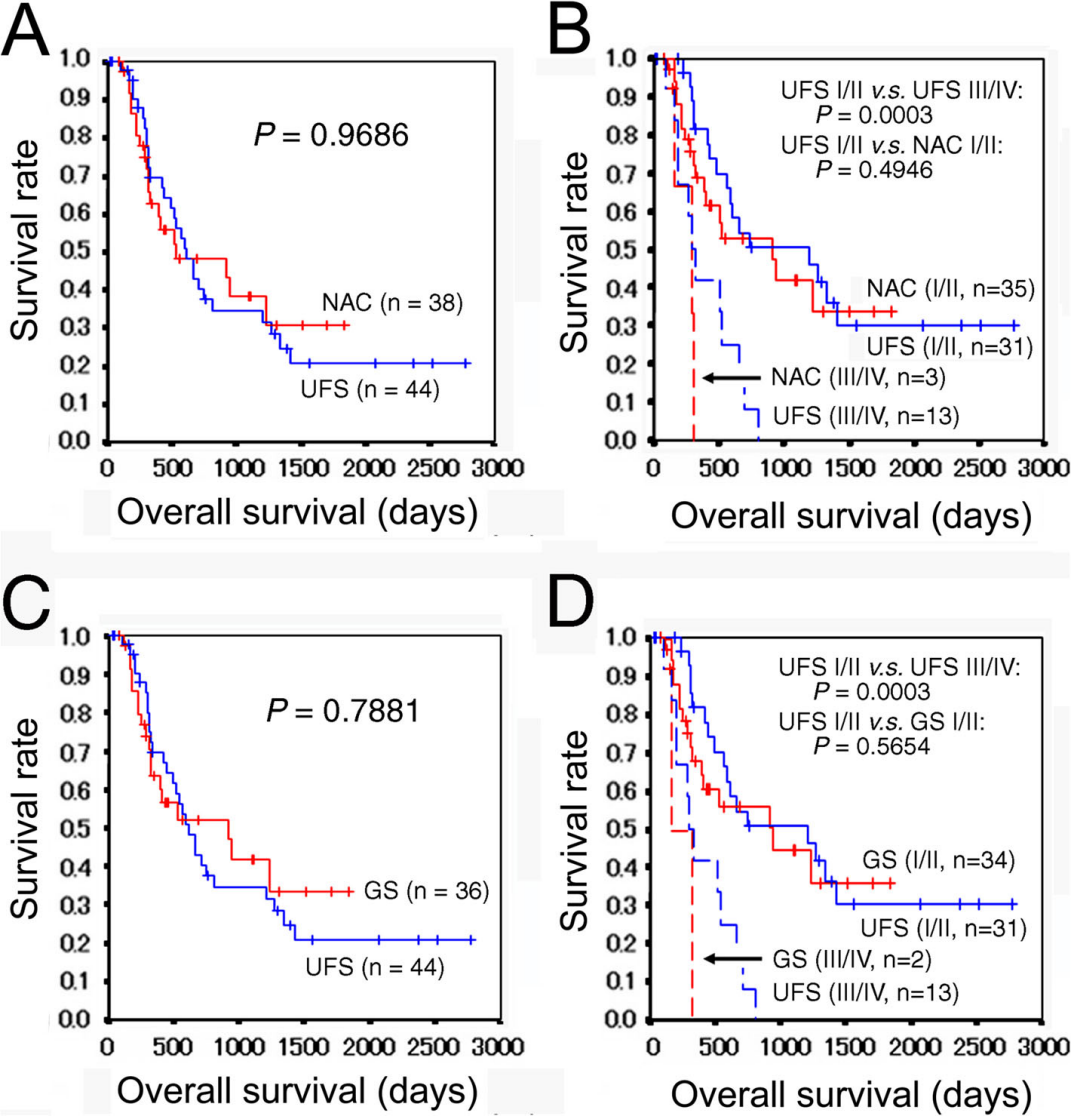
Overall survival curves of PDAC patients who did not receive postoperative chemotherapy with CDDP plus gemcitabine or S-1, GnP, or FOLFIRINOX. **a** Overall survivals of the UFS and NAC groups. **b** Comparison of the overall survivals between the UFS and NAC groups adjusted for the pathologic stage. **c** Overall survival of the UFS and NAC (GS) groups. **d** Comparison of the overall survivals between the UFS and NAC (GS) groups adjusted for the pathologic stage. PDAC, pancreatic ductal adenocarcinoma; CDDP, cisplatin; S-1, tegafur/gimeracil/oteracil, GnP, gemcitabine plus nab-paclitaxel, FOLFIRINOX, 5-fluorouracil/leucovorin plus irinotecan plus oxaliplatin; UFS, upfront surgery; NAC, neoadjuvant chemotherapy; GS, gemcitabine plus S-1

Postoperative chemotherapy and radiotherapy may affect postoperative prognosis. In our study subjects, there were significant differences in the use of the CDDP plus gemcitabine or S-1, GnP, and FOLFIRINOX between the UFS and NAC groups. These regimens were administered to patients who experienced recurrence/metastasis as a second-line or later postoperative chemotherapy, except for six patients who received GnP as a first line. We therefore excluded 49 patients who received these regimes from overall survival analysis. The NAC group (*n* = 38) demonstrated somewhat better overall survival as compared with the UFS group (*n* = 44), but the difference did not reach a statistical significance (*P* = 0.9686) (Fig. 2a). Furthermore, the NAC group with stages I/II (*n* = 35) demonstrated similar overall survival with the UFS group with stages I/II (*n* = 31) (*P* = 0.4946) (Fig. 2b).

**Fig. 3:**
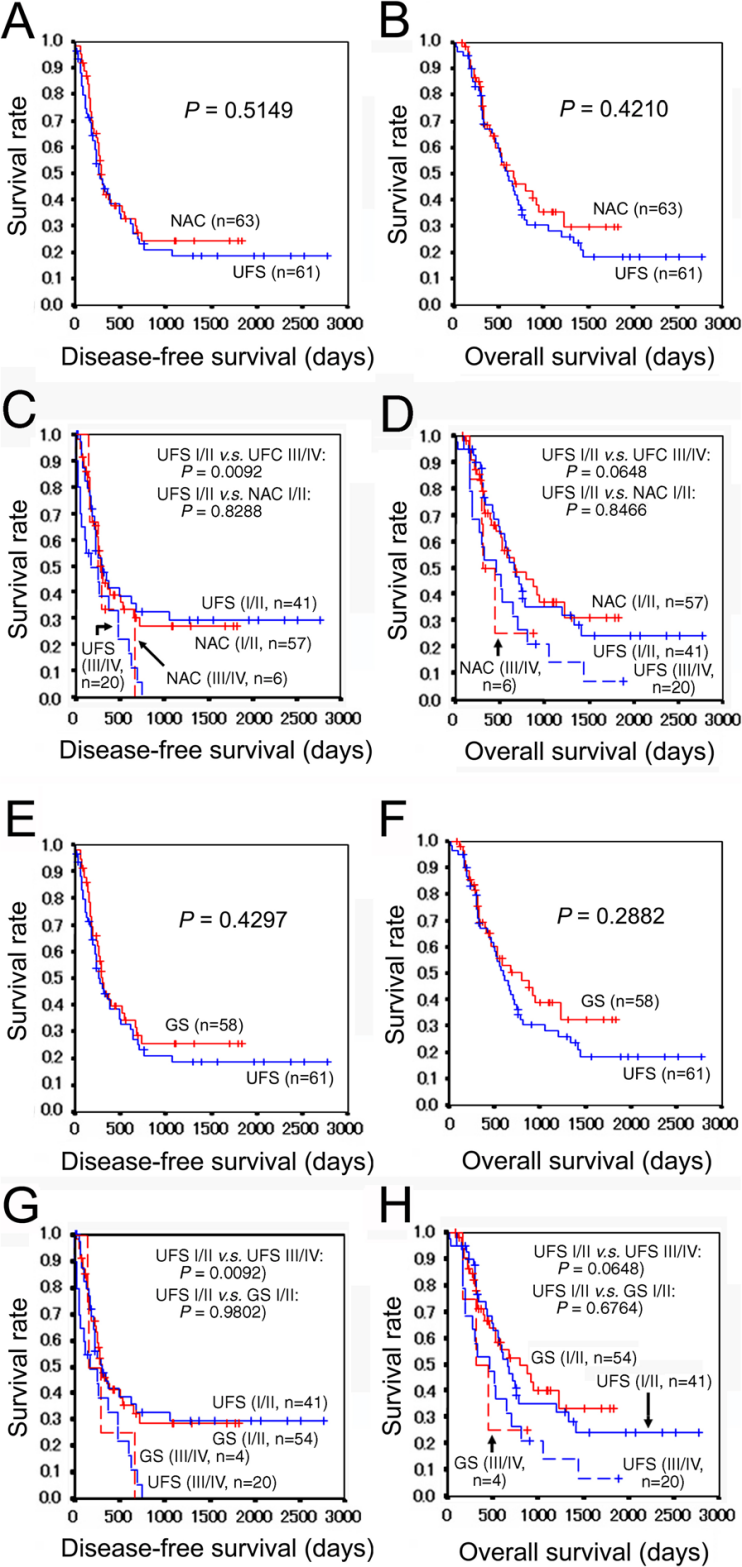
Survival curves of PDAC patients excluding patients who received postoperative radiotherapy. **a** Disease-free survivals of the UFS and NAC groups. **b** Overall survivals of the UFS and NAC groups. **c** Comparison of the disease-free survivals between the UFS and NAC groups adjusted for the pathologic stage. **d** Comparison of the overall survivals between the UFS and NAC groups adjusted for the pathologic stage. **e** Disease-free survivals of the UFS and NAC (GS) groups. **f** Overall survivals of the UFS and NAC (GS) groups. **g** Comparison of the disease-free survivals between the UFS and NAC (GS) groups adjusted for the pathologic stage. **h** Comparison of the overall survivals between the UFS and NAC (GS) groups adjusted for the pathologic stage. PDAC, pancreatic ductal adenocarcinoma; UFS, upfront surgery; NAC, neoadjuvant chemotherapy; GS, gemcitabine plus S-1 (tegafur/gimeracil/oteracil)

Then, survival analysis was performed after excluding seven patients with postoperative radiotherapy. The NAC group (*n* = 63) demonstrated somewhat better disease-free and overall survival as compared with the UFS group (*n* = 61), but the difference did not reach a statistical significance (*P* = 0.5149 and *P* = 0.4210, respectively) (Fig. 3a, b). Furthermore, the UFS group with stages I/II (*n* = 41) and the NAC group with stages I/II (*n* = 57) demonstrated similar disease-free survivals (*P* = 0.8288) and overall survivals (*P* = 0.8466) (Fig. 3c, d).

### Analyses of the effect of NAC specified to the GS regimen

Clinicopathologic findings and prognosis were compared between the UFS group (*n* = 64) and the NAC group specified to the GS regimen (*n* = 62). Tumor size was marginally smaller in the GS group than in the UFS group as shown by pT1 versus pT2/pT3 (*P* = 0.071). There were no significant differences in the differentiation grade and lymphovascular and neural invasions between the UFS group and the GS group. The degree of nodal metastasis was significantly lower (*P* = 0.005) and the rate of distant metastasis found at laparotomy was marginally lower (*P* = 0.058) in the GS group than in the UFS group. As a result, stages I/II were significantly more frequent than stages III/IV in the GS group compared with the UFS group (*P* < 0.001). Rate of R0 resection was marginally higher in the GS group than in the UFS group (*P* = 0.067), but there were no significant differences in R2 resection and surgery-related death between the UFS group and the GS group. Furthermore, there were no significant differences in the frequencies of overall postoperative chemotherapy and postoperative irradiation between the UFS group and the GS group. These results are summarized in Table 1.

Postoperative survivals were compared between the UFS group and the GS group. There were no significant differences between the two groups in disease-free survival curves (*P* = 0.4057) and overall survival curves (*P* = 0.2756), although overall survival in the GS group seemed somewhat better than that of the UFS group (Fig. 1e, f). Then, each group of patients was subdivided according to the pathologic stage, and prognoses of patients were compared with the stages adjusted. The UFS group with stages I/II (*n* = 43) and the GS group with stages I/II (*n* = 58) demonstrated similar disease-free survivals (*P* = 0.9425) and overall survivals (*P* = 0.7184) (Fig. 1g, h).

Postoperative chemotherapy and radiotherapy may affect postoperative prognosis. There were significant differences in the use of the CDDP plus gemcitabine or S-1 and FOLFIRINOX and marginally significant difference in the use of GnP between the UFS and GS groups (Table 2). These regimens were administered to patients who experienced recurrence/metastasis as a second-line or later postoperative chemotherapy, except for five patients who received GnP as a first line. We therefore excluded patients who received these regimen postoperatively from overall survival analysis. The GS group (*n* = 36) demonstrated somewhat better overall survival as compared with the UFS group (*n* = 44), but the difference did not reach a statistical significance (*P* = 0.7881) (Fig. 2c). Furthermore, the GS group with stages I/II (*n* = 34) demonstrated similar overall survival with the UFS group with stages I/II (*n* = 31) (*P* = 0.5654) (Fig. 2d).

Then, survival analysis was performed after excluding patients with postoperative radiotherapy. The GS group (*n* = 58) demonstrated somewhat better disease-free and overall survival as compared with the UFS group (*n* = 61), but the difference did not reach a statistical significance (*P* = 0.4297 and *P* = 0.2882, respectively) (Fig. 3e, f). The UFS group with stages I/II (*n* = 41) and the GS group with stages I/II (*n* = 54) demonstrated similar disease-free survivals (*P* = 0.9802) and overall survivals (*P* = 0.6764) (Fig. 3g, h).

## Discussion

More and more attention has been attracted to neoadjuvant therapy in the treatment of PDAC for the last decade. Many researchers have reported the effect of neoadjuvant therapy on various stages of PDAC. For example, Tang et al. reported that a complete response and a partial response were observed in 2.8% and 28.7% of patients with borderline resectable PDAC, respectively, whereas about 45.9% of patients showed a stable disease and 16.9% of patients developed tumor progression after neoadjuvant chemo- and/or radiotherapy [5]. Gillen et al. reported that average complete/partial response rates to neoadjuvant chemo- and/or radiotherapy were 3.6%/30.6% and 4.8%/30.2% for initially resectable tumors and initially non-resectable tumors, respectively, whereas progressive disease was estimated to 20.9% and 20.8% [18]. They also reported that resectability after neoadjuvant therapy was estimated to be 33.2% in initially unresectable tumors and 73.6% in initially resectable tumors [18]. Thus, around one-third of initially unresectable tumor might become resectable following neoadjuvant therapy, with comparable survival as those with resectable tumors [18]. Mokdad et al. and Sugimoto et al. reported in their retrospective studies that neoadjuvant therapy was associated with improved rates of R0 resection and a decreased incidence of lymph node metastasis in patients with resectable or non-metastatic PDAC [7, 19]. These studies suggest the efficacy of neoadjuvant treatment, which is indicated for resectable, borderline resectable, and locally advanced PDAC. Shaib et al. also reported that borderline resectable PDAC that received NAC even demonstrated more favorable prognosis than resectable PDAC that underwent upfront surgery [20]. In the present study, pT1 (primary tumor size of ≤ 2.0 cm) was marginally more frequent, degree of nodal metastasis was significantly lower, and rate of distant metastasis was significantly lower at laparotomy in the NAC group. These data may also suggest the therapeutic effect of NAC. As a result, stages I/II were significantly more frequent in the NAC group than in the UFS group. As a matter of fact, we cannot know the true pathologic stage of PDAC before NAC without exploratory laparotomy. Therefore, it cannot be excluded that NAC group may have had fewer stages III/IV in the first place. Out of the 42 patients who underwent surgery before 2014 when NAC was not common in our facilities, 27 cases were stages I/II and 15 were stages III/IV. This data and data of the UFS group (stages I/II, 43; stages III/IV, 21) may suggest that approximately two-thirds of our patients are stages I/II and one-third of our patients are stages III/IV. If this speculation is right, 61 of stages I/II and 6 of stages III/IV in the NAC group may suggest a positive effect of the NAC.

The R0 resection rate was marginally higher in the NAC (GS) group than in the UFS group. R2 resection was noted in a patient who underwent NAC, which was not statistically significant. As for perioperative complication rates following NAC, Deig et al. reported no significant differences in major perioperative complication rates or postoperative mortality in patients with potentially resectable PDAC who received neoadjuvant therapy versus UFS [21]. Hank et al. reported that neoadjuvant therapy might be associated with a significant reduction in the rate of postoperative pancreatic fistula but that once fistula occurred, it was associated with a significant reduction in long-term survival [22]. In our study, surgery-related death was noted in two patients who underwent UFS, which also was not statistically significant. Furthermore, Schorn et al. reported that neoadjuvant therapy may affect histopathologic features such as microvascular and lymphatic invasion, perineural invasion, and pathologic differentiation grade [23]. However, a significant difference was not observed in any of them between the NAC group and the UFS group in this study.

We then drew the Kaplan-Meier survival curves of the patients. We expected a better prognosis of the NAC group than the UFS group, which would be due to the therapeutic effect of NAC and/or the exclusion of aggressive tumor that did not respond to NAC, or both. Although the NAC group revealed somewhat better overall survival than the UFS group, a statistical significance was not demonstrated between the groups in disease-free and overall survivals. Similar results were obtained when excluding patients who received postoperative chemotherapy regimen of which the frequency was statistically different between the UFS and NAC groups. We speculate that definition of survival time might have adversely affected the NAC group, because, in this study, survival time initiated from day of surgery and duration of NAC was not included in the survival time. To remove this and other biases, prognosis of patient populations adjusted for initial clinical stage and other clinicopathologic parameters must be compared starting at the time of initial diagnosis. Another explanation is a low response rate of the NAC regimen. Two cycles of the GS regimen, which was used in the Prep-02/JSAP05 study, has been a standard regimen for NAC in Japan [13]. In this study, 92.5% of NAC were the GS regimen. Suzuki et al., a research group directed by Kubota who is also a member of this research group, reported that radiographic response to the GS therapy was 15.4% of partial response and 84.6% of stable disease in primary resectable PDAC [17]. All GS group (*n* = 38) and 27 of the UFS group (*n* = 37) in their prospective randomized control study are also included in this study subjects. We speculate that this low response rate might have diluted the effect of NAC on postoperative survival.

Meanwhile, it may be possible that there is really a smaller difference in postoperative prognosis between the UFS and NAC groups. Although not statistically significant, overall survival was more favorable in the NAC group than in the UFS group, but disease-free survival was not. In addition, although not statistically significant, patients tended to be younger in the NAC group than in the UFS group (Table 1). Patient’s age, which was not the case with disease-free survival, was a marginally significant prognostic factor of overall survival [24]. Therefore, patient’s age may have affected the overall survival in the later follow-up period.

The UFS and NAC groups demonstrated similar postoperative prognoses after adjustment of the pathologic stage. Similar results were obtained when excluding patients who received postoperative radiotherapy or who received postoperative chemotherapy regimen of which the frequency was statistically different between the UFS and NAC groups. We cannot know the true pathologic stage of PDAC before NAC without exploratory laparotomy. Assuming that GS-mediated downstaging from stage III/IV to stage I/II occurred in 20% of the original stage III/IV patients in the GS group based on the previous study [17], it is calculated that original stage I/II and III/IV patients were 57 and 5, respectively. This suggests that almost all GS group with pathologic stages I/II at surgery (57 of 58: 98.3%) might have been originally pathologic stages I/II before starting GS. In addition to downsizing and downstaging of the tumor (visible effects), NAC was intended to treat micro-metastasis and devitalize PDAC prior to surgery (invisible effects). However, the disease-free survival was not improved in the NAC group as compared with the UFS group after adjustment for pathologic stages. Distant metastasis is closely associated with cancer-associated death [24]. As a result, overall survivals were also similar between the groups. These results suggest that micro-metastasis and aggressiveness of the tumor may not be controlled in those tumors, comprising approximately 70 to 80% of the whole PDAC, which do not show radiological response to NAC. Another explanation may be NAC-induced chemoresistance. Adjuvant chemotherapy is the standard of care following surgical resection, with numerous studies showing improved long-term survival of patients treated with adjuvant chemotherapy [25]. In this study, 54 (84.4%) patients in the UFS group and 59 (88.1%) of the NAC group received postoperative chemotherapy including adjuvant chemotherapy. Out of the 62 patients who received NAC of the GS regimen, 8 patients first received gemcitabine, 43 patients first received S-1, and one patient first received GS as adjuvant chemotherapy. NAC of the GS regimen may have diminished the effect of adjuvant chemotherapy by inducing drug resistance and could not improve the postoperative prognosis as compared with the UFS group.

Prognosis of PDAC is significantly improved only by surgical resection. Resectability after neoadjuvant therapy was 73.6% in initially resectable tumors, while resection rates are 78–96% in patients with resectable tumors that are explored without neoadjuvant treatment [18]. Considering this report and our data that suggest limited invisible effects of NAC, UFS would be desirable for resectable PDAC. In contrast, NAC may be recommended to borderline resectable and locally advanced PDAC, because 80% of PDAC at least may not progress and 30% of locally advanced PDAC may become resectable after NAC.

To overcome the present difficulty, regimen for NAC should be reconsidered. FOLFIRINOX and GnP are currently considered the two best chemotherapy regimens for borderline resectable and locally advanced patients [26]. At present, FOLFIRINOX with/without modification and in combination with other reagents and irradiation may be the most favorable NAC regimen, but those regimens are so toxic that few patients can tolerate [27–30]. In addition, many neoadjuvant clinical trials for PDAC are currently running throughout the world [31]. It may be desirable that the NAC regimen and the adjuvant chemotherapy regimen do not overlap to avoid drug resistance. In addition, we expect analyses of respective regimens for evaluating not only visible effects but also invisible effects such as repressing micro-metastasis and devitalizing tumor cells as we did in the present study.

In conclusion, NAC may be useful in downstaging and improving prognosis in a small subset of PDAC, but effects of NAC, both visible and invisible, may not be remarkable in the majority of PDAC, at least with respect to the GS regimen. Postoperative prognosis may be determined at the pathologic stage of resected specimen with or without NAC. Therefore, it may be recommended that NAC should be applied to borderline resectable and locally advanced PDAC, while UFS would be desirable for primary resectable PDAC.

## Data Availability

The datasets used and analyzed in the current study are available from the corresponding author on reasonable request.

## Abbreviations

UFS: Upfront surgery
NAC: Neoadjuvant chemotherapy
PDAC: Pancreatic ductal adenocarcinoma
SMV: Superior mesenteric vein
PV: Portal vein
R0: Microscopically invasive cancer-free at all margins
CT: Computed tomography
R1: Microscopically involved by invasive cancer at any margin
S-1: Tegafur/gimeracil/oteracil
GS: Gemcitabine plus S-1
GnP: Gemcitabine plus nab-paclitaxel
FOLFIRINOX: 5-Fluorouracil/leucovorin plus irinotecan plus oxaliplatin
R2: Macroscopically residual tumor at any margin
